# Bone metabolism in children with normal weight and overweight/obesity in a northeastern region of Spain

**DOI:** 10.1515/almed-2024-0026

**Published:** 2024-03-08

**Authors:** José Cuenca Alcocel, Lorena Villalba-Heredia, Inés Martínez Redondo, Clara Berrozpe-Villabona, José Antonio Casajús, José Miguel Arbonés-Mainar, Pilar Calmarza

**Affiliations:** Service of Clinical Biochemistry, 16765 Obispo Polanco Hospital, Teruel, Spain; GENUD Research Group, 16765 University of Zaragoza, Zaragoza, Spain; Service of Pediatrics, 16488 Miguel Servet University Hospital, Zaragoza, Spain; Service of Preventive Medicine, 16488 Miguel Servet University Hospital, Zaragoza, Spain; GENUD (Growth, Exercise, Nutrition and Development) Research Group, 123305 University of Zaragoza, Healthcare Research Institute of Aragon (IIS Aragón), Zaragoza, Spain; Biomedical Research Networking Center for Physiopathology of Obesity and Nutrition (CIBEROBN), Carlos III Health Institute, Madrid, Spain; Department of Physiatry and Nursing, Faculty of Health and Sports Sciences, University of Zaragoza, Zaragoza, Spain; Adipocyte and Fat Biology Laboratory (AdipoFat), Transversal Research Unit, Miguel Servet University Hospital, Health Research Institute (IIS) Aragón, Zaragoza, Spain; Aragonese Institute of Health Sciences (IACS), Zaragoza, Spain; CIBER Pathophysiology Obesity and Nutrition (CIBERObn), Carlos III Health Institute, Madrid, Spain; Service of Clinical Biochemistry, 16488 Miguel Servet University Hospital, Zaragoza, Spain; Network Research Center for Cardiovascular Diseases (CIBERCV), University of Zaragoza, Healthcare Research Institute of Aragon (IIS Aragón), Zaragoza, Spain

**Keywords:** bone metabolism, overweight/obesity, bone turnover markers, children, correlation

## Abstract

**Objectives:**

Bone mass progressively increases to peak during childhood and adolescence, which determines future bone health. Bone formation–resorption processes are assessed using bone markers. However, studies on the impact of obesity on bone turnover markers at this age are limited, and results are inconsistent. The objective of this study was to examine the potential impact of overweight/obesity on bone metabolism.

**Methods:**

A study was performed to compare parameters of bone metabolism in 45 girls and boys with normal weight (controls) and in a group of 612 girls and boys with overweight/obesity (cases) from the Exergames study (University of Zaragoza). Ages ranged from 8 to 12 years.

**Results:**

Higher values of phosphorus and IGFBP-3 were observed in children with overweight/obesity, as compared to children with normal weight, (p=0.042) and (p=0.042), respectively. BAP, osteocalcin, magnesium, vitamin D and IGF-I concentrations were lower in the group with overweight/obesity, whereas calcium concentrations were higher in this group, although differences were not statistically significant. A negative correlation was found (r=−0.193) (p=0.049) between BAP and BMI.

**Conclusions:**

Although differences did not reach statistical significance, BAP and osteocalcin concentrations were lower in children with overweight/obesity. This added to the negative correlation found between BAP and MIC may demonstrate that overweight/obesity may negatively affect bone health already at a young age.

## Introduction

Obesity during childhood affects the onset of both, development and pubertal maturation, which may have an impact on early puberty and sexual development. Thus, obesity induces precocious puberty and early development of secondary sexual characteristics in the two sexes [1].

The prevalence of obesity in children and/or adolescents has increased in European countries in the last decades [2]. This condition is a serious public health problem worldwide and is associated with a higher risk for cardiovascular diseases and diabetes mellitus type 2 [3]. According to the ALADINO 2019 study [4] and other studies [5, 6], although the prevalence of obesity and overweight in children remains stable, it is very high, reaching 20 % in 6-to-12-year-old children.

The bone is a metabolically-active tissue that hosts bone formation and resorption processes, which occur simultaneously and at different sites [7]. During childhood and adolescence, bone metabolism increases to adapt to skeletal growth needs. As a result, bone mass increases to peak, which is known as “peak bone mass” [8], which determines future bone health. The age at peak bone mass varies as a function of different factors, including sex and genetic factors. In general, it is estimated that children reach their peak bone mass at 18–23 years [8]. A low bone mass involves a higher risk for fractures and predisposes subjects to bone diseases such as osteoporosis [9].

However, studies examining bone metabolism in children with obesity and overweight are limited, and results are inconsistent. In some studies, children and adolescents with overweight/obesity had a reduced bone mass [10]. In contrast, bone mineral density (BMD) was found in other studies to be higher in children and adolescents with overweight/obesity, as compared to children with normal weight [11]. In the former case, the impact of a higher BMD in childhood on bone health in adulthood is unknown.

Bone turnover markers are a series of substances produced during bone remodeling. These markers measure the products generated during the formation and degradation of the bone matrix, and can be determined either in serum and urine. Repeated measurements at short time intervals enable serial evaluation of bone turnover; moreover, they provide dynamic, fact-based information on skeletal health. In children, it is necessary to estimate growth rate and pubertal development to correctly interpret results [12]. Some of the most widely used markers of bone remodeling include osteocalcin and bone alkaline phosphatase (BAP), which indicate bone formation. Markers of bone resorption include β-CrossLaps (β-CTx) and C-terminal telopeptide of type-I collagen (CTx).

In relation to bone remodeling markers in children with overweight/obesity, results are inconsistent. In some studies, concentrations of bone markers were found to be lower in obese children, as compared to children with normal weight [13, 14]. In contrast, in other studies, concentrations of bone remodeling markers were observed to be similar in obese children and in children with normal weight [15].

Other studies provide evidence of an association between obesity and some inflammatory markers, such as interleukin-6 (IL-6), C-reactive protein (CRP), and tumor necrosis factor-α (TNF-α). Some of these markers reduced BMD, whereas others improved bone calcium build up [16].

The objective of this study was to evaluate parameters of bone metabolism in 8- to 12-year-old children with normal weight, as compared to children with obesity or overweight from Zaragoza. The sample of children with overweight/obesity was taken from the Exergames study cohort. Parameters of bone metabolism included bone remodeling markers, namely, osteocalcin and bone alkaline phosphatase (BAP), vitamin D, calcium, magnesium, insulin-like growth factor binding protein 3 (IGFBP-3), and somatomedin C (IGF-I). A secondary objective was to evaluate whether overweight/obesity influences these parameters.

## Materials and methods

An observational case-control study was performed in Zaragoza, Aragon, Spain. The control group was composed of 59 girls and boys with normal weight undergoing minor surgery (cryptorchidism, phimosis, trauma, among others). After a review of medical records, patients with confirmed disease and/or overweight/obesity identified according to the International Obesity Task Force (IOTF) criteria [17] were excluded.

The final control sample included 45 children.

The case group was composed of 61 girls and boys with overweight or obesity (estimated based on their BMI and according to the cut-off points established by Cole et al. [17], adjusted for age and height, with an equivalent BMI of 25 and 30 kg/m^2^ for overweight and obesity, respectively).

The case group was composed of children from the cohort of the Exergames study carried out by the University of Zaragoza.

**Figure 1:**
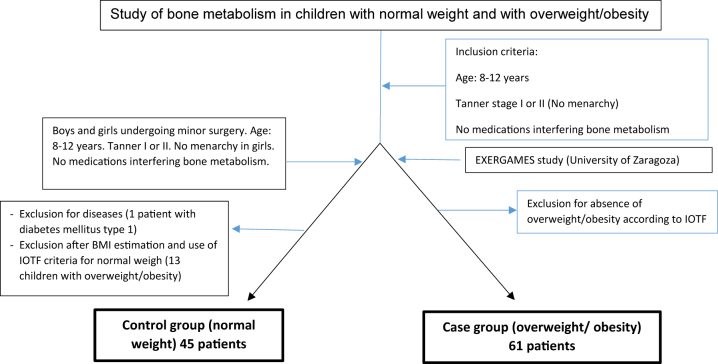
Selection of children with normal weight (control group) and children with overweight/obesity (case group).

The two groups included 8- to 12-year-old children living in the autonomous community of Aragon that had not experienced the onset of pubertal development, or menarche in girls (Tanner stages I and II). Children who were using vitamin D supplementation or had diseases or treatments that can influence the study parameters (bone, metabolic, chronic diseases, acute infection, anorexia nervosa) were excluded. Patient selection outflow is shown in Figure 1.

Parents received a patient information sheet and all signed the informed consent form. Then, a short questionnaire was administered to collect epidemiological and clinical data (Supplementary Material, Annex 1). Anthropometric data was collected [weight, height and body mass index (BMI)] and a physical examination was performed. Next, a fasting blood test was carried out to measure the parameters of interest and evaluate bone metabolism.

Blood samples were collected early in the morning after overnight fasting in serum-separator gel tubes. Calcium, phosphorus, and magnesium were measured in serum using spectrophotometric techniques on an AU 5800 autoanalyzer (Beckmann Coulter Miami, FL, USA). Osteocalcin, IGFBP-3, and IGF-I were determined automatically by electrochemiluminescence immunoassay on a Cobas e411analyzer (Roche Diagnostics, Spain). BAP concentration was determined using a manual ELISA assay followed by spectrophotometric reading. Vitamin D concentration was measured by automated immunoassays on an Architect i1000SR analyzer (Abbott Diagnostics, USA).

This study complies with all national regulations, institutional policies, and the ethical tenets of the Declaration of Helsinki and was approved by the Ethics Committee of the Autonomous Community of Aragon (CEICA).

Statistical analysis was performed with the IBM SPSS Statistics 26.0 software package.

Firstly, the Kolmogorov–Smirnov Lilliefors (KSL) test was used to examine the distribution of quantitative anthropometric variables.

Parametric quantitative variables (KSL, p>0.05) were presented as means and standard deviation, whereas non-parametric quantitative variables (KSL, p≤0.05) were expressed as median and interquartile range.

Normally-distributed anthropometric and biochemical variables were compared using Student’s t-test when variances in the two groups were homogeneous or Welck test for non-homogeneous variances. Differences in non-normally distributed variables were examined using Mann Whitney U test.

A similar statistical analysis was performed using the tests described above to determine the suitability of the two study groups in terms of age, height and BMI, assessing whether they were comparable.

To assess the correlation between biochemical parameters and BMI, age, and sex, the KSL test was first performed on the whole sample to assess the normality of variables. If the variable followed a normal distribution, Pearson coefficient of correlation was determined. In the case of dichotomous variables (as in the case of sex), Kendall’s tau-b test was performed. Level of significance was set at a p-value <0.05.

## Results

Anthropometric data and the most significant results of the questionnaire are shown in Table 1. No statistically significant differences were observed in terms of age between groups. The proportion of girls and boys, as well as results, was similar in the two groups (55.7 % of boys in the case group vs. 64.4 % in the control group, p=0.064). The number of bone fractures was also similar in the two groups, and only a boy in the case group had sustained repeated fractures (two fractures at different sites). Likewise, the prevalence of recurrent infections was similar in the two groups.

**Table 1:** Most significant anthropometric parameters of the questionnaire administered to children with normal weight and overweight/obesity.

	Overweight/obesity (n=61, 34 boys and 27 girls)			Normal weight (n=45, 29 boys and 16 girls)			Statistical significance (test)	Levene test p-value
	Total	Limits	Normality tests^c^	Total	Limits	Normality tests^c^		
Age, years	10.1±0.9^a^	(8.4–12.2)	0.200	10.1±1.1^a^	(8.4–12.0)	0.200	0.912 (Welch)	0.014
Weight, kg	55.4 (14.8)^b^	(33.4–89.1)	0.034	33.0±8.2^b^	(22.0–42.0)	0.200	<0.001 (U Mann–Whitney)	–
Size, cm	145±8^a^	(129–161)	0.200	138±9^a^	(119–155)	0.200	<0.001 (Student t)	0.521
BMI	25.8 (4.0)^b^	(20.1–36.0)	0.200	17.1±2.4^b^	(13.7–19.9)	0.023	<0.001 (Mann–Whitney U)	–

	**Median**	**Limits**		**Median**	**Limits**			

Weight Z-score	0.01	(−1.91–2.94)	–	0.13	(−1.91–1.80)	–	–	–
Size Z-score	−0.11	(−2.04–1.92)	–	0.09	(−2.24–1.96)	–	–	–
BMI Z-score	−0.02	(−1.75–3.01)	–	0.19	(−2.01–1.95)	–	–	–

	**Total**	**IC 95 %**		**Total**	**IC 95 %**			

Fractures (patient%)	18.0	(9.4–30.0)	–	11.1	(3.7–24.1)	–	0.325 (Pearson Chi squared)	–
Recurrent infections (patient%)	52.5	(39.3–65.4)	–	45.5	(30.4–61.2)	–	0.479 (Pearson Chi squared)	–

^a^Mean±standard deviation; ^b^median (interquartile range); ^c^Kolmogorov–Smirnov–Lilliefors test.

Laboratory results and statistical analysis test results are shown in Table 2.

**Table 2:** Type of distribution and comparative study of biochemical parameters in children with normal weight and children with overweight/obesity.

Parameters	Overweight/obesity		Normal weight		Statistical significance (test)	Levene test p-value
	Value	Normality tests^c^	Value	Normality tests^c^		
BAP, U/L	124.7±33.0 (116.2–133.1)^a^	0.200	134.0±32.2 (124.2–143.8)^a^	0.200	0.151 (Student t)	**0.467**
Osteocalcin, ng/mL	84.4; 37.2 (67.4–104.6)^b^	0.045	85.0; 36.8 (72.7–109.5)^b^	0.007	0.572 (U de Mann–Whitney)	–
Calcium, mg/dL	10.00; 0.5 (9.80–10.30)^b^	0.193	9.90; 0.5 (9.70–10.20)^b^	0.135	0.251 (U de Mann–Whitney)	–
Phosphorus, mg/dL	5.04; 0.69 (4.61–5.30)^b^	0.200	4.90; 0.60 (4.50–5.10)^b^	0.002	**0.042** (U de Mann–Whitney)	–
Magnesium, mg/dL	2.00; 0.25 (1.95–2.20)^b^	<0.001	2.10; 0.20 (2.00–2.20)^b^	0.003	0.349 (U de Mann–Whitney)	–
Vitamin D, nmol/L	59.8±18.7 (55.0–64.6)^a^	0.185	65.1±20.1 (59.1–71.1)^a^	0.052	0.165 (Student t)	**0.593**
IGFBP-3, μg/mL	5.69±1.27 (5.37–6.02)^a^	0.200	5.22±0.95 (4.93–5.51)^a^	0.200	**0.042** (Student t)	**0.061**
IGF-I, ng/mL	208.0; 109 (162.5–271.5)^b^	<0.001	216.0; 111 (159.0–270.0)^b^	0.007	0.866 (U de Mann–Whitney)	–

^a^Mean±standard deviation (95 % CI); ^b^median; interquartile range (Q1–Q3); ^c^Kolmogorov–Smirnov Lilliefors test. Bold values refer to the parameters where statistical significance tests showed a statistically significant difference between the two groups at a 95 % confidence level.

The group of children with overweight/obesity showed significantly higher phosphorus and IGFBP-3 concentrations, as compared to children with normal weight (p<0.042 and p<0.042, respectively). BAP, osteocalcin, magnesium, vitamin D and IGF-I concentrations were lower and calcium was higher in the case group. However, statistical tests revealed that concentrations were comparable between the two groups for a 95 % confidence interval.

There were statistically significant sex-based differences in BAP and IGF-1 concentrations, with girls showing higher values, p <0.05.

Girls with overweight/obesity showed significantly higher IGFBP-3 concentrations, p=0.026.

Boys in the case group showed significantly higher phosphorus concentrations (p=0.016) as compared to controls, with no statistically significant differences in girls.

**Figure 2:**
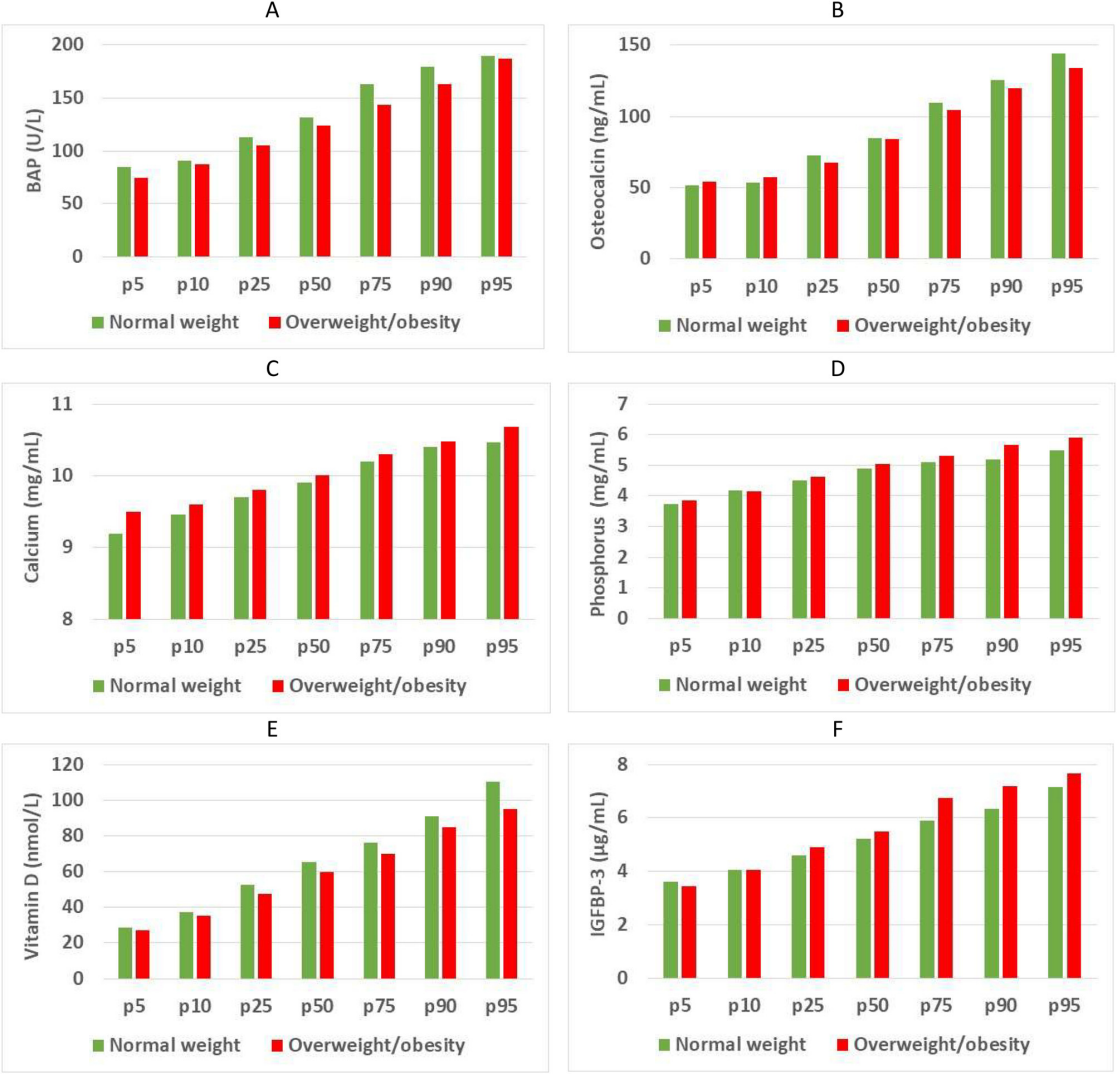
Percentiles of bone parameters of children with normal weight and children with overweight/obesity. (A) BAP, (B) osteocalcin, (C) calcium, (D) phosphorus, (E) vitamin D, (F) IGFBP-3, (G) IGF-I.

Figure 2 shows differences between groups in study parameters by percentile.

The correlation between the different biochemical parameters and BMI, age and sex are shown in Table 3. BMI was found to be negatively correlated with BAP and positively correlated with IGFBP-3. Additionally, a positive correlation was observed between age and BAP and osteocalcin. In girls, there was a positive correlation between female sex and BAP, IGFBP-3 and IGF-I.

**Table 3:** Correlation of biochemical parameters with age, sex and body mass index.

Parameter	Significance of normality test^a^	Correlation with BMI		Correlation with age		Correlation with sex^b^	
		Correlation coefficient r	Statistical significance	Correlation coefficient r	Statistical significance	Correlation coefficient r	Statistical significance
Age	0.200	–	–	–	–	–	–
IMC	<0.001	–	–	–	–	–	–
BAP	0.200	**−0.193**	**0.049**	**0.235**	**0.016**	**0.184**	**0.022**
Osteocalcin	0.002	−0.011	0.911	**0.247**	**0.011**	0.137	0.090
Calcium	0.142	0.115	0.240	0.030	0.759	0.013	0.872
Phosphorus	0.106	0.146	0.135	−0.094	0.340	0.056	0.495
Magnesium	<0.001	−0.136	0.166	−0.043	0.659	−0.021	0.814
Vitamin D	0.060	−0.130	0.184	−0.095	0.333	0.060	0.455
IGFBP-3	0.200	**0.276**	**0.005**	0.169	0.086	**0.241**	**0.003**
IGF-I	<0.001	0.079	0.428	0.384	<0.001	**0.189**	**0.019**

^a^Kolmogorov–Smirnov Lilliefors test;^b^if the correlation is positive, it means that the value of the parameter is greater in girls than in boys, whereas a negative correlation means greater values in boys. Bold values represent those parameters where the correlation coefficient is statistically significant at a 95% confidence level.

**Figure 3:**
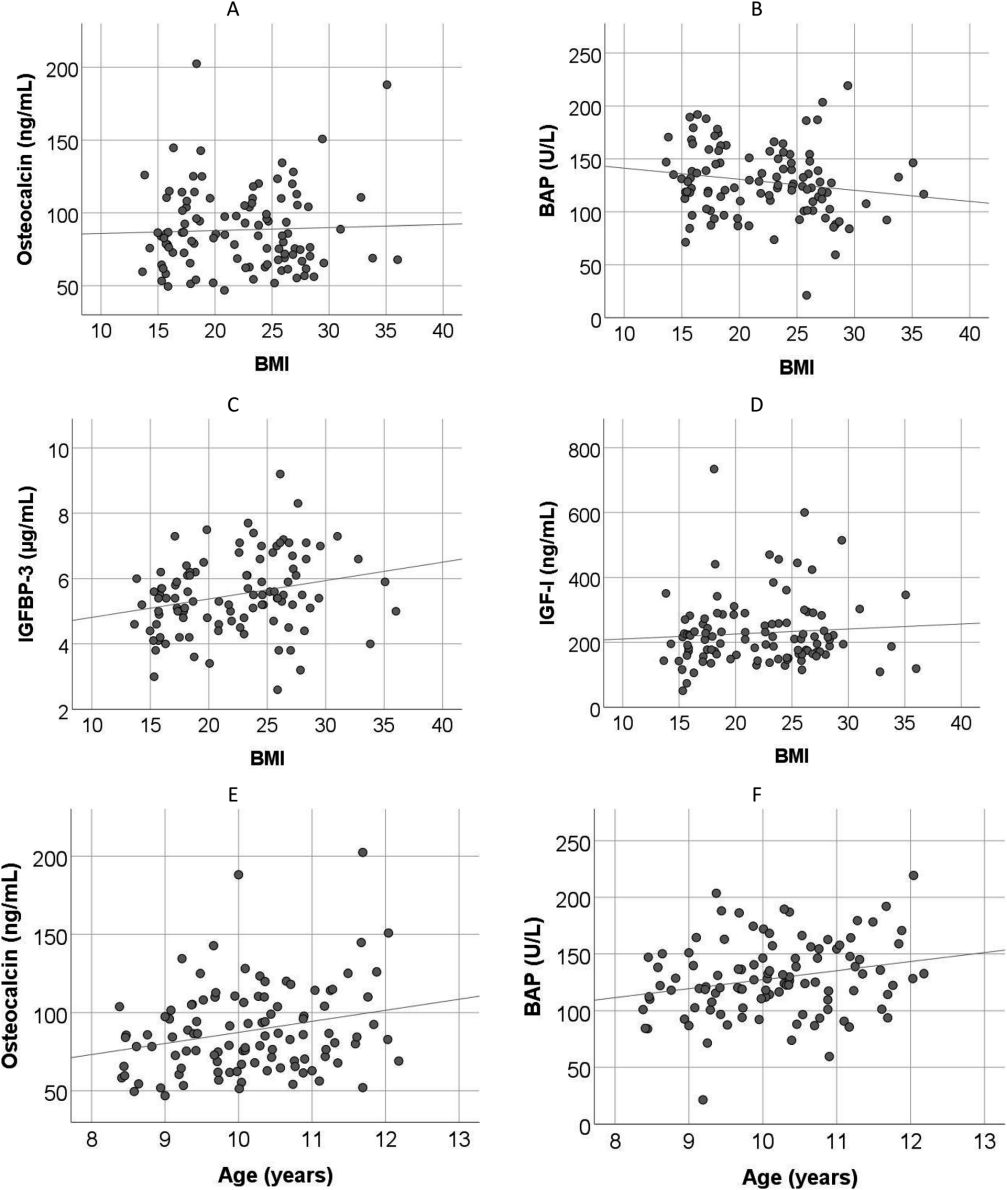
Correlation of bone metabolism parameters and IGFBP-3 with BMI and age. (A) Osteocalcin, (B) BAP, (C) IGFBP-3, (D) IGF-I, all with respect to BMI, (E) osteocalcin and (F) BAP, both with respect to age.

Figure 3 describes the correlation between BMI and BAP, osteocalcin, IGFBP-3 and IGF-I, and between age and osteocalcin and BAP.

## Discussion

Reaching an adequate bone mass in childhood prevents osteoporosis in adulthood. In addition, some prospective studies reveal an association between muscle fat and reduced muscle function, which may negatively affect growth. For this reason, understanding the impact of obesity on bone metabolism is of great interest.

Evidence has been provided that children with overweight/obesity have a higher bone mineral density than their counterparts with normal weight. However, some studies reveal an increased risk for limb fractures in children with overweight/obesity, which suggests poor bone quality [18].

In children with overweight/obesity, bone quality is influenced both, by inflammatory immunomodulatory cytokines and by the mechanical overload of the bone, as adipose and bone tissue are metabolically active, clearly-interconnected tissues [19]. Likewise, there is experimental evidence in animal models revealing that replacement of bone marrow with adipose tissue promotes low-grade inflammation, thereby reducing osteoblastic activity and promoting osteoclastic activity.

To evaluate bone formation, we selected BAP and osteocalcin, as they are some of the most widely-used markers of this process. Consistently with the results obtained by Saber et al. [15] in children of 7.55±3.34 years, our study showed that osteocalcin concentrations were similar in the two groups, and revealed higher phosphorus concentrations in boys with overweight/obesity. In the study by Mosca et al. [20], no statistically significant differences were found in osteocalcin concentrations between children of 10–13 years with normal weight and children with overweight/obesity.

In contrast, other authors report lower concentrations of bone markers in children with overweight/obesity, as compared to children with normal weight [13, 14]. Other studies reveal higher concentrations in obese children [21].

In our study, differences only reached statistical significance in phosphorus and IGFBP-3 concentrations, which were higher in children with overweight/obesity. However, these children also showed lower BAP, osteocalcin, magnesium, vitamin D and IGF-I concentrations, although differences were not statistically significant.

Phosphorus concentrations were higher in the case group, as compared to controls (p<0.042). Around 10 % of children in the case group had values above the upper limit of normality (3.7–5.6 mg/dL, for ages of 4–11 years) [22], whereas all controls showed concentrations within the range of reference.

The higher phosphorus and IGFBP-3 concentrations observed in children of 8–12 years with overweight/obesity indicate a higher growth rate in these children. Indeed, children in the case group had a higher height than controls.

Notably, no statistically significant differences were observed between the two groups in calcium and vitamin D concentrations, which are associated with phosphorus concentrations [23].

The evidence provided in the literature on calcium concentrations is inconsistent. Thus, some studies show higher calcium concentrations in obese children [24], whereas others report no significant differences [25].

In the two studies, vitamin D concentrations were lower in obese children, as compared to children with normal weight. A meta-analysis performed in 2020 revealed that the relative risk for an association between obesity and vitamin D deficiency was 1.41 (95 % CI: 1.26–1.59) [26]. This finding suggests that children and adolescents with overweight and obesity are at a higher risk for vitamin D deficiency. In our study, although differences were not significant between the two groups, controls exhibited slightly higher vitamin D concentrations in all percentiles, as shown in Figure 3. In relation to the association between bone markers and BMI, most studies exclude a correlation between osteocalcin and BMI [15, 27], which is consistent with our results. In agreement with our study, Cao et al. [28] documented in 2022 a negative correlation between BAP and BMI.

The evidence available about the association between bone metabolism in children with overweight/obesity is limited and, in some cases, inconsistent. On the one hand, Gajewska et al. conducted a study in 2015 [29] and found that IGF-I was higher in obese children, as compared to children with normal weight; moreover, total IGFBP-3 concentrations were similar in the two groups. In contrast, another study carried out in 2021 by Czogala et al. [30] showed similar IGF-I concentrations between groups, which is consistent with our results.

As mentioned above, the evidence available on bone markers in children with normal weight and with overweight/obesity is limited. Further studies are required to shed light on the controversial results available in the literature and in our study. Longitudinal studies would help identify changes in the correlation between bone markers and adipose tissue when children with normal weight gain too much weight. These studies would also be useful to determine the underlying cause of changes in the hormones that regulate these processes.

Apparently, calciotropic hormones, which finely regulate mineral metabolism, are influenced by hormonal factors directly involved in energy metabolism. There is robust evidence on the interrelationship between leptin, calciotropic hormones and bone turnover markers. These associations demonstrate the major role that adipose tissue plays in the skeleton and bone metabolism.

We expect to perform a larger study that separates children with overweight from children with obesity. Our research group also plans to measure some hormones that may be related to obesity to assess their influence on bone metabolism. Some of these hormones include leptin, the parathyroid hormone (PTH), and bone resorption markers (β-CTX or ICTP), among others.

In conclusion, children with overweight/obesity showed higher phosphorus and IGFBP-3 concentrations, as compared to children with normal weight. This means a higher growth rate in the former, although differences did not reach statistical significance. BAP and osteocalcin concentrations were lower in the case group. This added to the negative correlation observed between BAP and BMI may indicate that overweight/obesity may have deleterious effects on bone health already at a young age.

## Supplementary Material

Supplementary Material Details
