# Supplementary material for: Bone metabolism in children with normal weight and overweight/obesity in a northeastern region of Spain
Source: Adv Lab Med. 2024 Mar 8;5(1):66–74. doi: 10.1515/almed-2024-0026 (PMC11019894; doi:10.1515/almed-2024-0026)
Supplement: Supplementary file 1 — Supplementary Material Details [file j_almed-2024-0026_suppl_001.docx]

**Questionnaire administered to study participants**

- **AGE**:

- **SEX**:

- **MONTH OF BLOOD TEST (SEASON):**

**- WEIGHT/Z-SCORE: BMI/ Z-SCORE:**

**- SIZE/ Z-SCORE:**

- **PREVIOUS DISEASES**, especially, diseases related to bone metabolism:

o Fractures. How may? Site.

o Background treatment: anticonvulsants, glucocorticoids

o Infections.

o Diseases related to the immune system.

o Glucose level abnormalities, diabetes mellitus in the kid.

- **FAMILIAL HISTORY** (especially, bone diseases, cardiovascular disease or metabolic diseases)

- **PROPHYLAXIS:**

o Do you receive prophylactic treatment with vitamin D? YES / NO
